# Carcinoembryonic Antigen Expression and Resistance to Radiation and 5-Fluorouracil-Induced Apoptosis and Autophagy

**Published:** 2016-05-17

**Authors:** Ebrahim Eftekhar, Hajar Jaberie, Fakhraddin Naghibalhossaini

**Affiliations:** 1 Department of Biochemistry, Shiraz University of Medical Sciences, School of Medicine, Shiraz, Iran.; 2 Autoimmune Research Center, Shiraz University of Medical Sciences, School of Medicine, Shiraz, Iran.; ¥ Current address: Molecular Medicine Research Center, Hormozgan University of Medical Sciences, Bandar Abbas, Iran.

**Keywords:** Carcinoembryonic antigen (CEA), colorectal cancer, 5-fluorouracil, apoptosis, autophagy

## Abstract

Understanding the mechanism of tumor resistance is critical for cancer therapy. In this study, we investigated the effect of carcinoembryonic antigen (CEA) overexpression on UV-and 5-fluorouracil (5-FU)-induced apoptosis and autophagy in colorectal cancer cells. We used histone deacetylase (HDAC) inhibitor, NaB and DNA demethylating agent, 5-azacytidine (5-AZA) to induce CEA expression in HT29/219 and SW742 colorectal cancer cell lines. MTT assay was used to measure IC_50_ value of the cells exposed to graded concentrations of 5- FU with either 0.1 mM NaB or 1 μM 5-AZA for 72 h . Using CHO- and SW742-CEA transfectants, we also investigated the effect of CEA expression on UV- and 5-FU-induced apoptosis and autophagy. Treatment of HT29/219 cell line with NaB and 5-AZA increased CEA expression by 29% and 31%, respectively. Compared with control cells, the IC_50_ value for 5-FU of NaB and 5-AZA-treated cells increased by 40% and 57%, respectively. Treatment of SW742 cells with NaB or 5-AZA increased neither CEA expression nor the IC_50_ value for 5-FU. In comparison to parental cells, CEA expression also significantly protected transfected cells against UV-induced apoptosis. Decreased proportions of autophagy and apoptosis were also observed in 5-FU treated SW742- and CHO-CEA transfectants. We conclude that CEA expression can effectively protect colorectal cancer cells against radiation and drug-induced apoptosis and autophagy.

Understanding the mechanisms of tumor resistance is important for the early prediction of treatment efficacy and adjusting the anticancer modalities. Carcinoembryonic antigen (CEA, CEACAM5), first described by Gold and Freeman in 1965, is a member of the immuno-globulin gene superfamily overepressed by about 90% of colorectal cancers (CRCs) (1), and in other types of neoplasm, including gastrointestinal, breast and lung carcinoma (2). Serum CEA, the most widely used tumor marker for the management of CRC (3), has been reported to be associated with poor tumor response to chemoradiotherapy and an increased risk of relapse (4, 5). Previous findings of *in vitro* and *in vivo* studies have also suggested that CEA overexpression has an instrumental role in human cancer progression by inhibiting cell differentiation and anoikis, a form of apoptosis caused by detachment from extracellular matrix (6, 7).

The current standard treatments of malignant CRC tumors include surgical resection, chemo-therapy, and/or radiation therapy. 5-fluorouracil (5-FU) has been one of the most important phar-macological agents in CRC patients’ treatment for several decades. The compound, as an antimeta-bolite, inhibits thymidine biosynthesis and leads to cell cycle arrest and apoptosis. Resistance to anticancer drugs such as 5-FU is a common cause of failure in CRCchemotherapy (8). Although 5-FU efficacy has been improved in combination with other adjuvant drugs such as oxaliplatin or irinotecan, however, tumor cell resistance to chemotherapy and radiotherapy is one of the most important obstacles to cancer treatment in advanced CRC. Previous studies provided some evidence showing that transfection of human CRC cells by CEA cDNA augments resistance to 5-FU cytotoxicity in *in vitro* model (9, 10).

While apoptosis is considered as type I cell death program, autophagy is the second important type of physiological cell death which is referred as type II cell death. 5-FU chemotherapy induces both apoptosis and autophagy in colon cancer cells (11-13). It has been suggested that there is cross-talk between these two types of cell death processes (14, 15). Autophagy is believed to play an important role in tumor formation by functioning as a self-defense mechanism protecting cancer cells against chemotherapy-induced apoptosis (11, 16).

In this study, we have investigated the effect of CEA overexpression on resistance to UV and 5-FU- induced autophagy and apoptotic cell death. Insight in the molecular mechanisms of drug resistance can facilitate the identification of biological markers to predict drug response and therefore improve tumor-selective therapeutic strategy.

## Materials and methods

**Cell lines and transfection**

The human colorectal carcinoma cell lines SW742, HT29/219 and the Chinese hamster ovary (CHO) cell line were obtained from the National Cell Bank of Iran (NCBI, Pasteur Institute, Tehran). All cell lines were cultured in RPMI 1640 supplemented with 10% FBS, 2 mM glutamine, 100 U/ml penicillin, and 100 μg/ml streptomycin (all from Gibco) in a humidified 5% CO2 atmosphere at 37 °C. CHO and SW742 cells were stably transfected with full length CEA cDNA using pCDNA3.1 (+) expression vector (Invitrogen, USA), as described previously (9). The CEA protein content of transfected cell lines was determined by commercially available ELISA kit (CanAg Diagnostics AB, Gothenburg, Sweden) and the corresponding band of CEA (180 KD) was shown using western blot. 

**Cell treatment**

HT29/219 and SW742 cells at a density of 2.5 x 10^5^ cells were seeded in 6-cm tissue culture dishes under normal culture condition for 24 h. Cells were treated with an increasing concentration of sodium butyrate (NaB) (0, 0.1, 0.2, 0.5 and 1mM) or 5-azacytidine (5-AZA) (0, 0.125, 0.25, 0.5, 1 and 2 μM) for 10 and 72 h, respectively. Cell lysate was prepared as previously described (9) and the CEA content of cells was determined using CEA ELISA kit (CanAg Diagnostics AB, Gothenburg, Sweden). Cell viability was determined by MTT dye reduction assay as described previously (9). The concentration of NaB (0.1 mM) and 5-AZA (1 μM) that had no cytotoxic effect and induced higher level of CEA expression was selected to determine the effect of CEA overexpression on 5-FU resistance. Cells were simultaneously co-treated with NaB (0.1 mM) or 5-AZA (1 μM) and different concentrations of 5-FU (0, 0.78, 1.56, 3.125, 6.25, 12.5, 25 and 50 μM) for 72 h. MTT assay was used to determine the IC_50_ of 5-FU treatment.

**Ultraviolet irradiation of CEA-transfected cells**

CEA-transfected cell lines (CHO and SW742) were seeded at 2×10^5^ cells per 60 mm cell-culture petri dishes and grown to subconfluence. For UV irradiation, cells were exposed in PBS on ice to UVC (220 nm) generated by a 30 W UVC light source (Philips Inc, Netherland).The intensity of UVC light was measured using a UV meter (Leybold Didactic, GmbH). The cells were irradiated with 0.5 W/m^2^ of UVC light for 5 min. The viability of cells was measured by trypan blue staining. In all cases, irradiations were performed in triplicate and non-irradiated cells used as controls.

**Apoptosis analysis**

For the apoptosis assay, propideum iodide (PI) staining and DNA fragmentation were used as described previously (17). Briefly, CEA-transfected and control CHO and SW742 parental cells were seeded at a density of 3x10^5 ^cells in 10 cm tissue culture dishes. After 24 h, CHO and SW742 cells were exposed to 250 μM and 400 μM 5-FU, respectively. After 72 h of 5-FU treatment, cells were trypsinized, and fixed in 70% ethanol for 2 h. All PI-staining experiments were performed using both attached and the floating cells. Cells were resuspended in 1 ml DNA staining solution (20 μg/ml PI and 0.2 mg/ml RNase A in PBS) in dark on ice. DNA content of the cells was analyzed by flow cytometry analysis (FACScan, Becton Dickinson Immunocytometry Systems,San Jose, CA, USA) with an acquisition of 20,000 events. The sub-G1 peak was used as an indication of the percentage of apoptosis-induced cell death.

Nucleosomal DNA fragmentation was also analyzed to confirm the occurrence of apoptosis. After 5-FU treatment, soluble DNA was extracted from both floating and attached cells as follows. Cells were harvested and lysed in DNA lysis buffer (10 mM Tris-HCL pH 7.4, 0.2% Triton X-100, 10 mM EDTA). After RNAse A (100 μg/ml) treat-ment, samples were incubated at 37 °C for 2 h and DNA was extracted with phenol: chloroform: isoamyl alcohol. The extracted DNA was preci-pitated with sodium acetate at final concentration of 300 μM and 2 volumes of 100% ethanol at -20 °C for 24 h. DNA sample was electrophoresed on a 2% agarose gel and stained with GelRed™ (Biotium Inc., USA). DNA bands were visualized under UV illumination.

**Autophagy detection by acridine orange staining**

For autophagy analysis, the development of acidic vesicular organelles (AVO) was quantified as described previously (18). For this purpose, CEA-transfected CHO and SW742 and control parental cells were seeded in 6-cm tissue culture dishes at a density of 3x10^5^ and incubated for 24 h. After treatment with 250 μM (for CHO) and 400 μM (for SW742) 5-FU for 72 h, cells were stained under normal culture condition with acridine orange at final concentration of 1μg/ml for 15 min. The acridine orange was removed and fluorescent micrographs were taken using an inverted fluorescent microscope equipped with a digital camera.

**Statistical analysis**

Data from multiple experiments were expressed as mean ± SD. Differences between groups were tested by Kruskal-Wallis test and Mann-Whitney *U* test. P< 0.05 was considered to be significant.

## Results

**NaB and 5- AZA induce CEA expression and 5-FU resistance**

Previous studies have demonstrated that histone deacetylase (HDAC) inhibitor NaB and the DNA methyltransferase inhibitor 5-AZA induces CEA expression in tumor cell lines (19-21). To induce upregulation of CEA in HT29/219 and SW742 cell lines, we treated these cells with different concentrations of NaB and 5-AZA. We first examined the cytotoxicity of various concentrations of NaB (0, 0.1, 0.2, 0.5 and 1mM) and 5-AZA (0, 0.125, 0.25, 0.5, 1 and 2 μM) on cancer cells. NaB and 5-AZA were not cytotoxic at 0.1 mM and 1 μM, respectively. At higher concentrations, cell growth was significantly inhibited (data not shown).Therefore, 0.1 mM concentration of NaB and 1 μM 5-AZA were used for all subsequent experiments.

Treatment of HT29/219 cell line with 0.1 mM NaB for 10 h and with 1 μM of 5-AZA for 72 h increased CEA expression by 29.2% and 31%, respectively (Tables 1 and 2). However, CEA expression in SW742 cell line was not affected by drugs even at higher concentrations used (i.e. 1 mM of NaB and 2 μM of 5-AZA). To investigate the effect of CEA overexpression on 5-FU resistance, HT29/219 and SW742 cells were co-treated with NaB (0.1 mM) or 5-AZA (1 μM) and graded concentration of 5-FU (0.39, 0.78, 1.56, 3.125, 6.25, 12.5, 25, 50, and 100 μM) for 72 h. The IC_50_ value for 5-FU treatment in NaB or 5-AZA-treated HT29/219 cells measured by MTT assay, were 12.1± 3.4 μM (8.67± 3.2μM in controls) and 13.65± 4.2 (8.67± 3.2 μM in controls), respectively (Tables 1 and 2). On the other hand, treatment of SW742 cells with NaB or 5-AZA increased neither CEA expression nor the IC_50_ value for 5-FU (Tables 1 and 2).

**Table 1:** Sodium butyrate (NaB)-induced CEA expression in HT29/219 and SW742 cells and resistance to anticancer drug 5-FU**.**

**Cell line**	** NaB ** **(mM)**	**CEA expression level** **(ng/mg protein)**	**IC**_50_ ** of 5-FU (μM)** ^a^ **Mean± SD**	^*^ **P-value**
HT29/219	0 0.1	20.2± 0.3 28.5± 3.3	8.67± 3.2 12.1± 3.4	0.04
SW742	0 0.1	0.00 0.00	21.9± 8.4 19.3± 3.2	0.26

a Results are presented as mean± SD of three independent experiments, each done in triplicate.

* P-value from Fisher’s exact test.

**Table 2:** 5-azacytidine (5-AZA)-induced CEA expression in HT29/219 and SW742 cells and resistance to anticancer drug 5-FU .

**Cell line**	**5-AZA** **(μM)**	**CEA expression level** **(ng/mg protein)**	**IC**_50_ ** of 5-FU (μM)** ^a^ **Mean± SD**	^*^ **P-value**
HT29/219	0 1	20.2± 0.3 29.2± 4.2	8.67± 3.2 13.65± 4**.**2	0.01
SW742	0 1	0.00 0.00	21.9± 8.4 20.5± 5.2	0.33

a Results are presented as mean± SD of three independent experiments, each done in triplicate.

* P-value from Fisher’s exact test.

**Table 3:** The effect of UV irradiation on viability of CEA transfected cell lines.

**Cell line**	^a^ **CEA expression level** **(ng/mg protein)**	^a^ **% viability after UV** **irradiation**	^*^ **P-value**
CHO CHO/CEA	0.00 70.3± 1.2	72.8± 6.7 81.7± 3.7	0.01
SW742 SW742/CEA	0.00 13.02± 0.9	38.2± 11.8 70.8± 11.4	0.001

^a^Results are presented as mean±SD of three independent experiments, each done in triplicate.

^*^P-value from Fisher’s exact test.

**CEA protects colorectal cancer cells against UV irradiation**

We also investigated the effect of CEA expression on resistance of cells against UV irradiation, a physical inducer of apoptosis. We previously established stable CEA transfectants of CEA-negative CRC cell line SW742 as well as CHO cells by transfection with full length CEA cDNA (9). After transfection, CEA protein expression levels measured in total cell lysates in CHO and SW742 transfectants were 70 and 13.02 ng of CEA protein per mg total protein content at sub-confluent stage, respectively (Table 3). Semi-confluent cell culture of CEA-transfectants and control untransfected cells were treated with 0.5 W/m^2^ UVC light and cell viability was determined by trypan blue staining. Both CHO- and SW742-CEA transfectants were more resistant to UV induced cell death than control parental cells (Table 3). In comparison to the control groups, CEA expression in CHO and SW742 cells conferred 10.8 and 46% resistance against UV induced cytotoxicity, respectively.

**CEA protects cells from 5-FU induced apoptosis**

**Fig 1:**
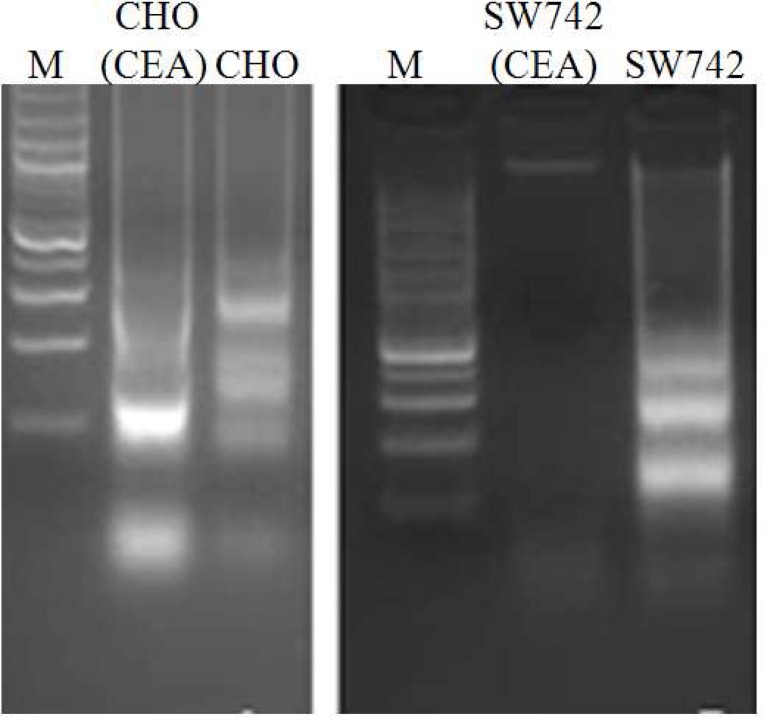
DNA fragmentation assay for apoptosis detection. Agarose gel electrophoresis of fragmented cellular DNA induced by 5-FU treatment. M: 100 bp DNA size marker; CHO, SW742: control parental cells; CHO (CEA), SW742 (CEA): CEA transfected cells.

**Fig 2:**
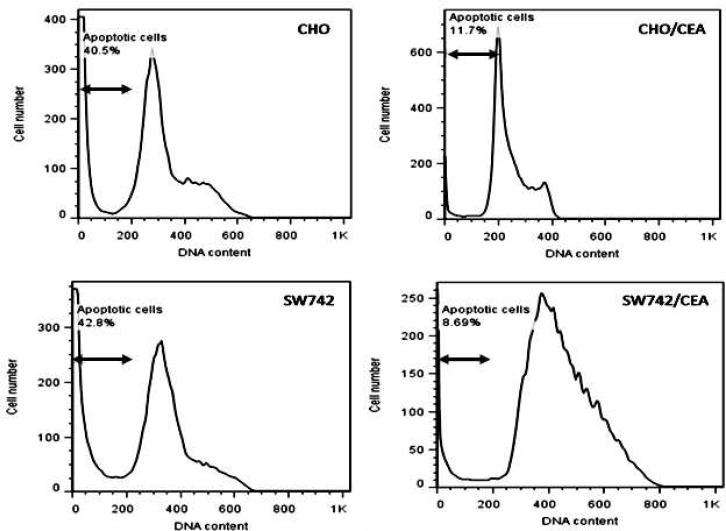
The histogram of DNA content distribution of CEA tranfected CHO and SW742 and control parental cells. Cells were treated with 5-FU for 72 h, fixed in ethanol, stained with propidium iodide, and DNA content was determined by flow cytometry. The arrowhead marks the apoptotic peak, namely sub-G1 peak in these cells. CEA transfectants show significantly lower apoptotic rate than their control counterparts.

Apoptosis has been implicated as one of the mechanism of cell death induced by 5-FU in colon cancer cells (12,16, 22). Thus, we decided to examine if CEA overexpression was capable to protect cells against 5-FU induced apoptosis. CEA-transfected CHO and SW742 cells were incubated for 72 h with 5-FU at concentrations of 250 and 400 μM, respectively. Electrophoresis of nuclear DNA was performed to observe DNA laddering, a hallmark for apoptosis, in 5-FU-treated cells. Notably, the extent of DNA fragmentation was greater in control parental cells than CEA transfected cells (Fig. 1). Flow cytometric analysis using propodium iodide staining and flow cytometry was also performed to quantify apoptosis of 5-FU-treated cells. Compared with the control parental cells, CEA transfected CHO and SW742 cells had a significantly lower apoptotic rates (71% and 79% reduced apoptosis, respectively) (Fig. 2).

**Overexpression of CEA protects cells from 5-FU induced autophagy**

**Fig 3:**
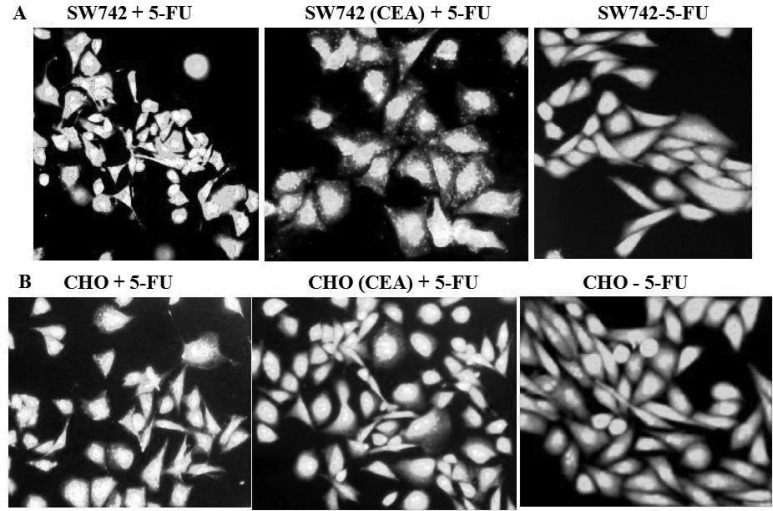
CEA inhibits autophagy induced by 5-FU. A) SW742- and B) CHO-CEA transfectants as well as control parental cells were treated with 5-FU for 72 h. After staining with acridine orange, cells were examined by fluorescence microscopy. The right figure in both panels is 5-FU-untreated parental cells. Acridine positive, bright fluorescent-stained vacuoles showed significant increase in autophagic cells in 5-FU treated parental cell populations, but very few in CEA-transfected cells. Photomicrographs are the representatives of three independent experiments.

Authophagy is an alternative type of programmed cell death that is activated in human colon cancer cells after treatment with 5-FU (11, 23). Using two CEA-transfected cell lines, SW742 and CHO, we investigated the effect of CEA expression on 5-FU induced autophagy. 5-FU treated cells were stained with acridine orange. As shown in Figure 3A and 3B, 5-FU treatment increased the acridine orange– positive cells with higher bright fluorescence, which has been reported to be a specific marker for acidic autophagic vacuoles (24). However, the number of autophagy positive cells in CEA- transfected CHO and SW742 cells were markedly less than that of their parental control counterparts. We conclude that CEA expression could protect cells against 5-FU induced autophagy. To our knowledge, the protective effect of CEA against 5-FU induced autophagy has not been reported before.

## Discussion

5-FU has been the drug of choice for the treatment of CRC patients for several decades. However, many of CRC patients have tumors intrinsically resistant to 5-FU-induced cytotoxicity. Previous work in our laboratory has shown that increased stable expression of CEA in cells was associated with increased resistance against 5-FU (expressed as higher IC_50_ value) (9). The objectives of the present study were to further investigate how changes in the levels of CEA expression could increase resistance against chemical and physical-induced cytotoxicity.

It has been reported that HDAC inhibitor, NaB and DNA methyltransferase inhibitor, 5-AZA upregulate CEA expression in different cancer cells (19-21). Therefore, we examined the effect of NaB and 5-AZA on 5-FU-induced cytotoxicity in two CRC cell lines. HT29/219 and SW742 cells were treated with different concentrations of NaB and 5-AZA. 10 h incubation with 0.1 mM NaB and 72 h incubation with 1 μM 5-AZA had no cytotoxic effect and induced high level of CEA expression in HT29/219, and were therefore used to determine the effect of CEA overexpression on 5-FU resistance in these cells. In HT29/219, NaB and 5-AZA increased 5-FU resistance by 28% and 36% compared with the control untreated cells, respectively (P< 0.05) (Tables 1 and 2). 5-AZA and NaB treatment did not increase CEA expression in CEA-negative SW742 cells. The CEA gene in this cell line is possibly disrupted by mutational events like deletion and the lack of CEA expression in this cell line could not be reversed by epigenetic modifying agents, AZA and NaB.

Our findings are consistent with previous results, showing that drug-resistant cells from human colorectal adenocarcinoma tumors produce higher than normal levels of CEA per cell (25, 26). Increased expression of CEA in colon carcinoma cells has been shown to be correlated with promoter hypomethylation of this gene in comparison to normal cells (27-29). NaB and 5-AZA appear to have major effects in increasing the rate of transcription through epigenetic modifications of chromatin and DNA methylation, respectively. Since inhibitors treatment changed neither CEA expression nor 5-FU resistance in SW742 cells, the results argue for a direct role of CEA for conferring drug resistance in cells expressing this protein rather than other effect or mechanisms mediated by inhibitors.

Our findings are in agreement with a previous report (10) showing that CEA expression can effectively protect cells against physical apoptosis inducing agent like UV irradiation. Using CEA-targeting ribozyme in HT29 colon cancer cells, Soeth et al. showed that CEA significantly protected HT29 cells from undergoing apoptosis under various stress conditions; including confluent growth and UV light (10). They also reported that CEA affects expression of various groups of cancer related genes, in particular cell cycle and apoptotic genes in HT-29 human CRC cells. The results obtained in the present study are consistent with other reports showing that 5-FU treatment induces both apoptosis and autophagy in cancer cells (12,16).

CHO and SW742 cell lines were transfected with CEA to investigate the effect of CEA overexpression on apoptosis and autophagy. Both CHO and SW742 cells are negative-CEA expressing cells. We used CHO as a control to verify the protective effects of CEA expression against 5-FU and radiation induced apoptosis and autophagy in other than CRC-derived cells. CHO- and SW742-CEA transfectants were treated with 250 and 400 μM 5-FU for 72 h, respectively.72 h drug exposure was chosen because a DNA-directed effect of 5-FU is observed when cells are exposed for a relatively long time (30, 31) and 5-FU is stable for this period of time in culture medium (32). While expression of CEA in CHO transfected cells was higher than transfected SW742, CHO cells showed less resistance to UV-irradiation (10% versus 46%) (Table 3). There are obviously many other factors other than CEA expression levels that may contribute to the radiation resistance. These include various genetic and epigenetic mechanisms involved in apoptosis, autophagy, and radiation-induced cell injury process (33).

We used DNA ladder assay and flow cytometric analysis of propidium iodide stained cells to assess apoptosis. We found that the relative frequency of apoptosis observed in the parental control cell lines, as verified by DNA laddering assay, was higher than those of CEA-transfected (5-FU-resistant cells). Flow cytometry analysis showed that CEA transfected cells were 70% more resistant against 5-FU-induced apoptosis than the parental cells (Fig. 1 and 2). The *in vivo* study by Bluementhal et al. showed that labetuzumab, the humanized anti-CEA antibody, can augment the therapeutic effects of 5-FU (34).

Our findings are in agreement with previous reports showing that autophagy is stimulated in response to chemotherapy (16, 35, 36). We analyzed the effect of CEA expression on the autophagic response to 5-FU. Staining with acridine indicated that the relative number of autophagic vacuoles in SW742- and CHO-CEA transfectants treated with 5-FU was significantly less than that of their control parental lines (Fig. 3). It appears that overexpression of CEA effectively increases resistance against 5-FU-induced autophagy.

The role of autophagy in tumorigenesis is controversial, because it can promote both cell death and survival of tumor cells (36, 37). It could also function as a tumor suppressor in the early stages of tumor development and as a protooncogene in advanced stages (38, 39).

The relationship between apoptosis and autophagy is also complex. Although, autophagy is an independent mechanism of cell death, there lies upstream of apoptosis and is necessary for the latter to occur (11, 16, 40).

Although, more sensitive methods such as quantitative measurement of acidic vesicular organelles by flow cytometry and western blot analysis of microtubule-associated protein light chain 3 (LC3) should be used to confirm our results, to our knowledge, this is the first study to demonstrate that CEA expression serves to protect against chemotherapy-induced autophagy.
